# Barriers to managing child and adolescent mental health problems: a systematic review of primary care practitioners’ perceptions

**DOI:** 10.3399/bjgp16X687061

**Published:** 2016-09-13

**Authors:** Doireann O’Brien, Kate Harvey, Jessica Howse, Tessa Reardon, Cathy Creswell

**Affiliations:** School of Psychology and Clinical Language Sciences, University of Reading, Reading.; School of Psychology and Clinical Language Sciences, University of Reading, Reading.; School of Psychology and Clinical Language Sciences, University of Reading, Reading.; School of Psychology and Clinical Language Sciences, University of Reading, Reading.; School of Psychology and Clinical Language Sciences, University of Reading, Reading.

**Keywords:** access to health care, barriers, child mental disorders, general practice, primary health care

## Abstract

**Background:**

Mental health problems are common and typically have an early onset. Effective treatments for mental health problems in childhood and adolescence are available, yet only a minority of children who are affected access them. This is of serious concern, considering the far-reaching and long-term negative consequences of such problems. Primary care is usually the first port of call for concerned parents so it is important to understand how primary care practitioners manage child and adolescent mental health problems and the barriers they face.

**Aim:**

To ascertain primary care practitioners’ perceptions of the barriers that prevent effective management of child and adolescent mental health problems.

**Design and setting:**

A systematic review of qualitative and quantitative literature in a primary care setting.

**Method:**

A database search of peer-reviewed articles using PsycINFO, MEDLINE^®^, Embase, and Web of Science, from inception (earliest 1806) until October 2014, was conducted. Additional studies were identified through hand searches and forward-citation searches. Studies needed to have at least one search term in four categories: primary care, childhood/adolescence, mental health, and barriers.

**Results:**

A total of 4151 articles were identified, of which 43 were included (30 quantitative studies and 13 qualitative studies). The majority of the barriers related to identification, management, and/or referral. Considerable barriers included a lack of providers and resources, extensive waiting lists, and financial restrictions.

**Conclusion:**

The identification of a broad range of significant barriers highlights the need to strengthen the ability to deal with these common difficulties in primary care. There is a particular need for tools and training to aid accurate identification and management, and for more efficient access to specialist services.

## INTRODUCTION

The majority of mental health problems start in childhood and adolescence,^1^^,^^2^ with 75% of adults with a mental health disorder experiencing the onset of the problem before the age of 24 years.^2^ Indeed, worldwide prevalence rates of mental health problems in children and young people have been estimated at 13.4%.^3^ The high prevalence of mental health problems,^4^ their negative impact on educational, occupational, and social functioning, as well as quality of life,^5^^–^7 and their significant financial and societal cost,^8^^,^^9^ emphasise the need for identification and effective treatment of mental health problems in children and young people.

Effective treatments for child and adolescent mental health disorders have been established in the developed world.^10^^,^^11^ However, there is a clear gap between prevalence and treatment rates, with only 25–35% of affected children and adolescents accessing treatment.^12^^–^17

Primary care practitioners play a key role in the recognition and management of child and adolescent mental health problems. Typically, the average British child sees their GP at least once a year^18^ (with similar patterns seen in other developed countries)^17^ and the GP is usually the first port of call for parents who are concerned about their child’s mental health.^19^^–^21 As such, primary care practitioners have the capacity to have a longstanding relationship with the family, and an understanding of the context of the family’s issues. Families highly value the input of these practitioners and welcome their involvement,^22^^,^^23^ which places them in a strong position to manage childhood mental health problems.

Government directives in developed countries have increasingly seen primary care practitioners as the ‘gatekeepers’ to young people’s mental health services.^24^^–^27 However, difficulties exist for primary care practitioners in both identification and management of mental health problems. For example, a recent study in the US found that primary care practitioners identified just 30% of children with a diagnosable depressive or anxiety disorder.^28^ Children and adolescents display symptoms of mental health problems in different ways from adults, may not be as forthcoming with their issues, and may more commonly present with physical symptoms.^29^^–^31 Indeed, a recent systematic review reported huge variability in the ability of paediatricians to recognise emotional and behavioural problems in primary care; it suggested that, overall, this skill was quite poor,^32^ particularly when the child’s problem is not severe.^33^ These problems are, no doubt, compounded by the fact that consultation time in primary care is typically short: patients in the UK discuss their mental health problems with a primary care practitioner for an average of 9 minutes per consultation.^34^

Primary care practitioners also face challenges once they have identified the presence of a mental health problem: only a minority of children and young people with diagnosed problems access specialist mental health services,^35^ and those who do get referred onwards often experience significant delays in receiving specialist help.^7^^,^^36^ Although some characteristics of patients who are more likely to be referred on from primary care have been identified — for example, majority ethnicity, higher parental perceived burden, greater symptom severity^14^^,^^37^^–^39 — little is known about why other children and adolescents are not accessing specialist help. Specifically, little is known about primary care practitioners’ perspectives on identifying and managing child and adolescent mental health problems in primary care, and primary care practitioners themselves have identified that their role in this area requires further research and definition.^40^ The aim of this systematic review, therefore, was to investigate and synthesise the available qualitative and quantitative literature pertaining to primary care practitioners’ experiences of barriers and facilitators to the effective management of child and adolescent mental health problems.

**How this fits in**

A significant number of barriers prevent primary care practitioners from effectively supporting children and adolescents with mental health problems. Difficulties with identification, time restrictions, and a lack of specialist mental health providers are major impediments. As well as providing an overview of barriers that primary care practitioners face when trying to manage these conditions, this review identifies areas of need, and makes recommendations for enabling improvements to strengthen the ability of primary care practitioners to deal with these conditions and to increase access to specialist services.

## METHOD

### Types of studies

This review, carried out according to Preferred Reporting Items for Systematic reviews and Meta-Analyses (PRISMA) guidelines,^41^ focused on primary care practitioners who have a ‘gatekeeper’ role to mental health services. Although their title may differ according to country (for example, GP, family physician, paediatrician), previous research suggests that common problems exist internationally regarding managing child and adolescent mental health problems.^42^

Studies were eligible if they involved eliciting primary care practitioners’ views of barriers or facilitators to the recognition and management of child and adolescent mental problems in primary care, and referral to specialist services. Where participants represented different professions, studies were included in which >80% of the sample were primary care practitioners. Barriers and facilitators were defined as primary care practitioner-perceived factors that promote or hinder the management of child and adolescent mental health problems. These factors had to have an explanatory value, which included primary care practitioners’ desired changes. All mental health problems were included, for example, eating disorders, self-harm, suicide, and attention deficit hyperactivity disorder (ADHD), as were studies that focused on mental health more broadly.

Pervasive developmental disorders and mental retardation (as defined in the text revision of the fourth edition of the *Diagnostic and Statistical Manual of Mental Disorders*) were excluded due to their treatability. Substance-use disorders were excluded as they are often treated outside of generic child and adolescent mental health services.^43^ Studies were also excluded if they:

- were not published in a peer-reviewed journal;
- were not available in English;
- were published before 1960;
- constituted a review, case study, or meta-analysis;
- had insufficient data to extract;
- specifically pertained to psychotropic medication;
- discussed a specific intervention or training course;
- were evaluating a specific tool;
- involved a population with a primary diagnosis other than a mental health problem (for example, cystic fibrosis, autistic spectrum conditions, or substance misuse); or
- looked at a specific patient population, for example, particular ethnic groups. These groups were considered to be likely to have specific needs and to access help through routes other than primary care (as highlighted in Cauce and colleagues^44^ and Bernal and colleagues^45^) and, as such, were beyond the scope of this review.

Children and adolescents were defined as patients aged <21 years, with a mean age of ≤18 years.

### Search strategy

A combination of search terms (Appendix 1) was used to ensure a high chance of capturing eligible studies. The strategy dictated that studies had to have at least one term in each of four categories relating to:

- practitioner type;
- children and adolescents;
- mental health problems; and
- barriers.

MEDLINE^®^, Embase, PsycINFO, and the Web of Science Core Collection were searched from inception (earliest 1806) until 30 October 2014. Reference lists of the final included studies were searched by hand and Web of Science was used to conduct a forward-citation search of all included studies.

### Selection of studies

Two authors independently screened all of the identified abstracts. A pilot test on a sample of 350 abstracts was conducted to ensure the criteria were fully understood by both, and to refine the inclusion/exclusion criteria. The exclusion criteria were hierarchical, with the first reason being the most important. Agreement between the two raters at abstract stage was moderate, with a kappa (κ) of 0.48 (95% confidence interval [CI] = 0.43 to 0.528). If a study was included by one or both of the authors, it was taken through to the full-text stage.

Following a further pilot test, all full texts were independently screened for inclusion by the same two authors in parallel. Agreement between the two raters at full-text stage was moderate (κ = 0.51, 95% CI = 0.385 to 0.64). When raters disagreed on whether to include a study, it was reviewed independently by a third researcher.

### Data extraction and management

Two authors independently extracted a standard set of data using a pre-specified form (Appendix 2). This included themes and quotes from the qualitative studies, and numerical data from the quantitative studies pertaining to explicitly described barriers or facilitators. Demographic data about the study and the sample were also extracted.

Each study was given a ‘contribution to the review’ score; this could be small, medium, or large, based on the amount of extracted data and how generalisable the results were to the overall review (that is, whether the study focused on a specific mental health problem or on mental health in general). Before final extraction, two researchers extracted data from 10% of the studies in parallel to check the data sheets were being used consistently. When discrepancies with extracted data were identified between the two researchers, these were discussed with a third researcher to achieve consensus.

### Assessment of methodological quality

Two authors independently assessed the quality of the quantitative studies using Kmet and colleagues’ checklist.^46^ Certain items that were not appropriate for the studies in this review were discarded, creating a 10-item list:

- Is the question/objective sufficiently described?
- Is the study design evident and appropriate?
- Is the method of participant selection described appropriate?
- Is the sample size appropriate?
- Are participant characteristics sufficiently described?
- Is the measure of barriers well defined?
- Is the measure of high quality/robust?
- Are analyses described/justified and appropriate?
- Are results reported in sufficient detail?
- Are the conclusions supported by the results?

For each item, the study was classified as:

- yes — study reached appropriate quality;
- partial — query was addressed, but not very thoroughly; or
- no — study did not resolve this item.

The first half of the checklist dealt with issues relating to the study as a whole, whereas the second half related to the specific data being extracted (that is, barriers/facilitator data).

For the qualitative studies, two of the authors assessed quality, using a nine-item checklist that incorporated questions from Kmet and colleagues^46^ and Dixon-Woods and colleagues’ prompts:^47^

- Is the question/objective sufficiently described?
- Are the research questions suited to qualitative inquiry?
- Is the study design well described and appropriate?
- Is the context of the study clear?
- Is the sampling strategy systematic, clearly described, and appropriate?
- Are the data collection methods clearly described, justified, and appropriate for the research question?
- Is the data analysis described, justified, and appropriate for the research question?
- Have verification procedures been used to establish credibility?
- Are the claims/conclusions credible and supported by evidence?

The procedure for rating the qualitative studies was the same as that for the quantitative studies.

Due to the heterogeneous nature of the studies in this review, quality was not used as an exclusion criterion. Discrepancies were resolved through a more collaborative process than in earlier phases, in which raters discussed issues to achieve consensus agreement for each item. Studies were then scored and classified as being of high, medium, or low quality:

- quantitiative studies: >7.5 = high quality, 5–7.5 = medium quality, and <5 = low quality; and
- qualitative studies: >7 = high quality, 7–4.5 = medium quality, and <4.5 = low quality).

### Data synthesis

The barriers and facilitators that were extracted were categorised as follows:

- recognition and diagnosis — issues specifically discussed surrounding recognition, identification, and diagnosis of a mental health issue;
- management — issues specifically discussed surrounding the management, treatment, and intervention of mental health issues;
- referral — issues specifically discussed surrounding referrals and issues associated with patients post-referral; or
- undifferentiated — could not be categorised into the above groups, as they did not clearly specify a stage of primary care management.

Within these categories, thematic analysis was used to group the data into themes. These themes were reviewed and discussed with the other authors in order to maximise reliability and credibility. Due to the heterogeneous nature of the quantitative data, it was not possible to derive overall scores for the emerging themes; instead, the barriers were labelled as low (<30% participants endorsed), medium (30–60% endorsed), or high (>60% endorsed). The number of studies that examined each barrier was represented graphically, organised by stage (recognition, management, referral, or undifferentiated).

Quantitative and qualitative data were synthesised to give a comprehensive picture of the information provided by the selected studies.

## RESULTS

### Study selection

**Figure 1.**
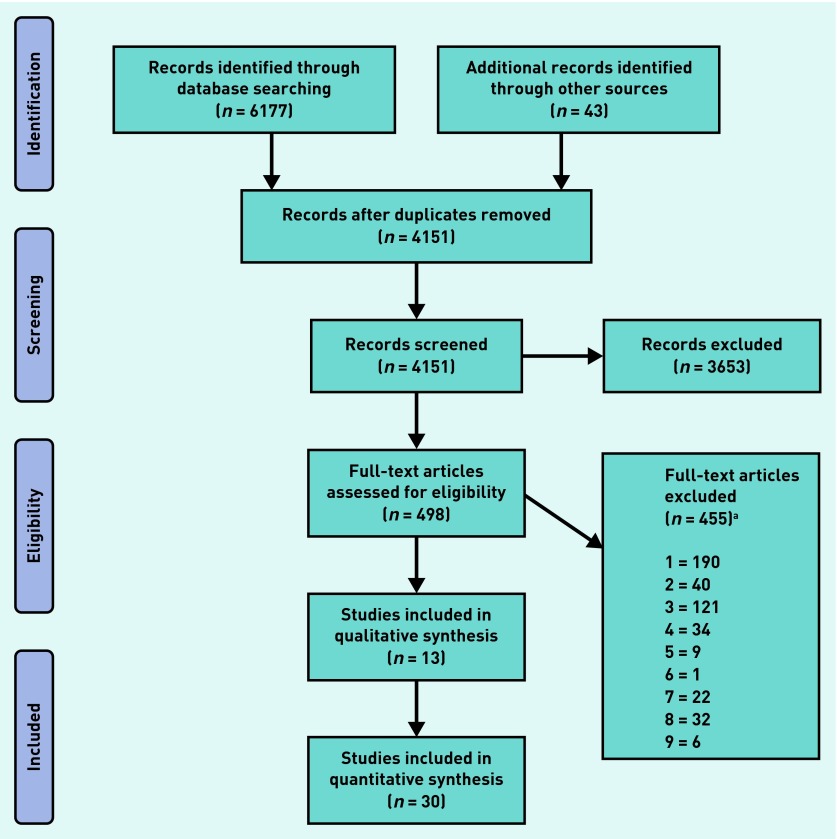
***Study selection.*** *^a^****Numbers 1–9 are the exclusion reasons. 1. Responders must be PCPs. 2. PCP must be reporting on a child and/or adolescent population. 3. PCPs must be reporting barriers/facilitators to management of mental health problems. 4. PCPs must be reporting on a mental health problems. 5. Peer-reviewed journal (for example, not books or dissertations) post-1960. 6. English language. 7. Must be able to extract data. 8. Exclude reviews, case studies, or meta-analyses. 9. Exclude studies focused on pervasive developmental/congenital disorders. PCP = primary care practitioner.***

The study selection process is shown in Figure 1. The database search identified 6177 studies; hand-searching and citation-searching of relevant articles unearthed a further 43 articles, then duplicates were removed, bringing the total to 4151. Following abstract screening, 498 remained for full-text examination. In total, 43 studies published between 1984 and 2014 satisfied the inclusion criteria, of which 30 were quantitative and 13 qualitative. All of the quantitative studies used survey data, whereas the qualitative studies were based on either one-to-one interviews or focus groups.

Twenty-two studies presented data from the US, with others from the UK (*n* = 9), Canada (*n* = 4), Australia (*n* = 4), Ireland (*n* = 2), South Africa (*n* = 2), Malta (*n* = 1), and Puerto Rico (*n* = 1). The majority of studies did not focus specifically on barriers and/or facilitators but dealt with wider aspects of primary care. Twenty-five of these studies pertained to mental health in general, and the other 18 focused on specific disorders such as: ADHD; post-traumatic stress disorder; suicidal behaviour; and mood, anxiety, and sleep disorders.

### Data quality and contribution to the review

Characteristics of the included studies^48^^–^90 are given in Table 1. Of the 13 qualitative studies, there was considerable variation in the quality: six were considered to be high-, four medium-, and three low-quality studies. There was also a spread in the quality of the quantitative studies with 17 studies rated as high, 10 medium, and three poor. Analyses to ascertain whether the poor-quality studies (three qualitative and three quantitative) were exerting an overt influence on the data indicated that these studies were not distorting or having a powerful impact on the overall themes. As such, all studies were retained.

**Table 1.** Study characteristics

**Author(s)**	**Year**	**Study design**	**Participants, *n***	**Country**	**Type of participant**	**Patient focus**	**Mental health focus**	**Quality assessment score**	**Contribution to the review**	**Notes**
Banh *et al*^73^	2008	Survey	546	US	Paediatrician	Child	Post-traumatic stress	High	Large	—
Bryce and Gordon^79^	2000	Questionnaire	348	Scotland	GP	Mixed	Mental health in general	Medium	Large	—
Faruqui *et al*^72^	2011	Questionnaire	346	US	Paediatrician	Mixed	Sleep disorders	High	Small	—
Goldberg *et al*^80^	1980	Encounter form completed after each patient visit	9	US	Paediatrician	Mixed	Mental health in general	Low	Small	—
Heneghan *et al*^66^	2008	Survey	132	US	Paediatrician	Child	Mental health in general	High	Large	—
Pidano *et al*^65^	2011	Survey	48	US	Paediatrician and family physician	Mixed	Emotional and behavioural problems	High	Large	—
Steele *et al*^63^	2010	Survey	106	Canada	Family physician and primary care paediatrician	Mixed	General mental health	Medium	Medium	Urban primary care practitioners Rural primary care practitioners
Taliaferro *et al*^61^	2013	Survey	387	US	Family practice and paediatrician	Adolescent	Depression	High	Large	—
Alexander and Fraser^81^	2008	Questionnaire	38	Australia	GP	Mixed	Mental health in general	High	Small	Children Adolescents
Horwitz *et al*^67^	2007	Survey	687	US	Paediatrician	Child	Psychosocial issues	High	Large	—
Lafrance *et al*^82^	2013	Survey	76	Canada	Family physician	Mixed	Eating disorders	High	Medium	Primary care practitioners with low self-assessed competence Primary care practitioners with high self-assessed competence
Louw *et al*^76^	2009	Questionnaire or structured interview for non-responders	229	South Africa	GP	Child	ADHD	High	Medium	—
McNicholas^74^	1997	Questionnaire	74	Ireland	GP	Child	Mental health in general	Medium	Medium	—
Olson *et al*^68^	2001	Survey	280	US	Paediatrician	Mixed	Depression	High	Large	—
Pidano *et al*^71^	2014	Survey	72	US	Paediatrician and nurse and physician‘s assistant	Mixed	Mental health in general	High	Large	—
Rushton *et al*^83^	2002	Child behaviour study survey	385	US, Canada, Puerto Rico	Paediatrician and family physician and GP	Mixed	Psychosocial problems	High	Medium	—
Shaw *et al*^84^	2002	Questionnaire	399	Australia	GP	Mixed	ADHD	Medium	Medium	—
Walders *et al*^85^	2003	Questionnaire	319	US	Paediatrician	Mixed	Mental health in general	High	Large	Managed care Fee for service
Venter *et al*^86^	2003	Questionnaire	143	South Africa	GP	Mixed	ADHD	Medium	Small	—
Frankenfield *et al*^77^	2000	Questionnaire	693	US	Paediatrician and family physician	Adolescent	Adolescent suicide	High	Medium	—
Goldberg *et al*^87^	1984	Survey	30	US	Paediatrician	Mixed	Mental health in general	Medium	Small	—
Healy *et al*^88^	2013	Survey (open- and closed-ended questions)	39	Ireland	GP	Mixed	Youth mental health	Medium	Small	—
Ross *et al*^62^	2011	Survey	100	US	Paediatrician	Mixed	ADHD, anxiety and depression	High	Medium	—
Rushton *et al*^69^	2000	Survey	591	US	Family physician and paediatrician	Mixed	Childhood depression	High	Small	Family physicians and paediatricians presented as one group
Rushton *et al*^70^	2004	Survey	723	US	Family physician and paediatrician	Child	ADHD	Low	Small	—
Veit *et al*^78^	1996	Questionnaire	687	Australia	GP	Adolescent	Health in general	High	Small	—
Williams *et al*^60^	2005	Standard interview with both multiple choice and open-ended questions	47	US	Paediatrician	Mixed	Behavioural health disorders	Medium	Small	—
Steele *et al*^63^	2012	Survey	847	Canada	Family physician and paediatrician and GP	Mixed	Mental health in general	Medium	Medium	—
Weeramanthri and Keaney^89^	2000	Questionnaire (delivered through interview)	20	England	GP	Mixed	Mental health in general	Low	Small	—
Mutale^75^	1995	Questionnaire	210	England	GP	Child	Mental health in general	Medium	Medium	—
Jones and Bhadrinath^59^	1998	Interview	47	England	GP	Mixed	Mental health in general	Low	Medium	—
Hinrichs *et al*^48^	2012	Interviews	7	England	GP	Mixed	Mental health in general	Medium	Medium	
DeSocio *et al*^51^	2007	Focus group	5	US	Paediatrician	Mixed	Childhood eating disorders	Medium	Medium	
Shaw *et al*^54^	2003	Focus group	28	Australia	GP	Mixed	ADHD	Medium	Medium	
Klasen and Goodman^52^	2000	Semi-structured interviews	10	UK	GP	Child	Hyperactivity	Medium	Small	
Pfefferle^56^	2007	Open-ended comments	596	US	Paediatrician	Child	Mental health in general	High	Large	
Richardson *et al*^57^	2007	Focus group	35	US	Paediatrician & paediatric nurse	Adolescent	Depression	High	Medium	
Salt *et al*^55^	2005	Semi-structured interviews	13	UK	GP	Child	ADHD	Low	Small	
Fiks *et al*^58^	2011	Semi-structured interviews	30	US	Paediatrician	Child	ADHD	High	Medium	
Roberts *et al*^53^	2014	Interviews	19	England	GP	Adolescent	Psychological difficulties	High	Small	
Williams *et al*^49^	2004	Structured standard interview	47	US	Paediatrician	Child	Behavioural health disorders	Low	Medium	
Roberts *et al*^50^	2013	Semi-structured interviews	19	England	GP	Adolescent	Emotional distress	High	Medium	
Buhagiar and Cassar^90^	2012	Questionnaire	157	Malta	GP	Mixed	Mental health in general	High	Small	

ADHD = attention deficit hyperactivity disorder.

Studies varied greatly in the extent to which they contributed to the review (Table 1): only one qualitative study made a large contribution, while eight made a medium contribution, and four a small one. Nine quantitative studies made a large contribution, 10 a medium one, and 11 a small contribution. Nonetheless, all studies were treated as equal in the analysis.

### Data extraction and summary of results

**Figure 2.**
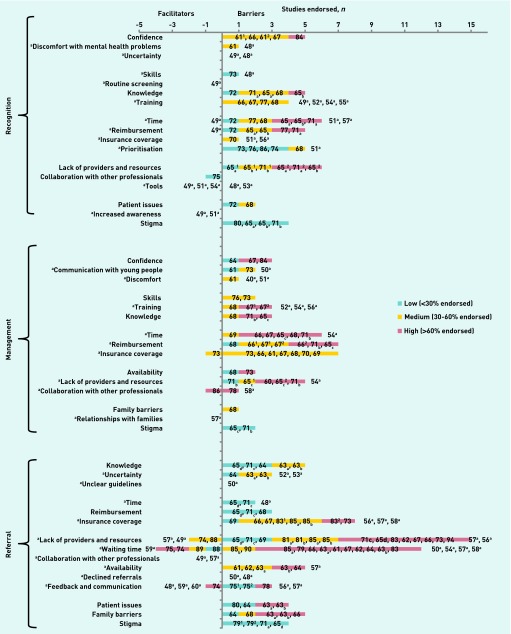
***Recognition, management, and referral barriers.*** ***Studies with no colour coding are qualitative (as denoted by the ‘a’) and, as such, level of endorsement does not apply. Superscript numbers mean that the study had more than one item querying this topic and subscript letters are related to the information provided in the Notes column of Table 1.***

Figure 2 provides an overview of the study findings at the following stages:

- recognition and diagnosis;
- management in primary care; and
- referral to specialist services.

Confidence, time, knowledge, reimbursement, and a lack of providers and resources posed the biggest barrier for primary care practitioners in recognising and diagnosing mental health problems in young people. Reimbursement, a lack of insurance coverage, time restrictions, and a lack of providers and resources posed significant barriers to primary care practitioners’ management of child and adolescent mental health problems. A lack of providers and resources (the most highly endorsed barrier overall), insurance coverage, waiting times, and availability of resources posed significant barriers to primary care practitioners’ referrals to specialist services as did patient issues and family barriers. Qualitative data for these sections is provided in Table 2.

**Table 2.** Summary of qualitative barriers and facilitators by diagnosis/treatment phase

	**Recognition and diagnosis**	**Management**	**Referral**
**Confidence**	Reluctance to ask *‘deeper’* questions^48^ Lack of clarity of diagnostic criteria, issues around potential comorbidity, parental discrepancies,^49^ children’s inability to express themselves well: *‘*[mental health problems] *don’t come to light so easily’*,^48^ lead to issues with confidence	Difficulties *‘establishing a rapport, finding the right words and tone to use and dealing with silence’*^50^ with younger patients Reluctance to broach the issue [of mental health] for fear of provoking *‘defensiveness and anxiety’* in the young person^51^	Uncertain where to refer^52^^,^^53^ — *‘Long, unhelpful letters from specialists’*^52^ Uncertainty regarding *‘the lack of clarity’* about how other services are structured and governed led to lack of confidence^51^
**Knowledge and skills**	Lack of emphasis on mental health in medical training^49^^,^^52^^,^^54^^,^^55^ ‘[re: hyperactivity] *you have to learn all about these diseases that have a prevalence of about one in a million, and this relatively common problem is hardly ever mentioned’*^52^ Lack of skills;^48^ it was suggested routine screening could increase	Lack of training:^52^^,^^54^^,^^57^ *‘My paediatric residency didn’t include adequate training for the amount of paediatric mental health problems there* [are] *in the world!’* 56	

**Prioritisation of mental health problems**	Lack of time to carry out exploratory screening^51^^,^^57^ More time needed for evaluation^49^ Increased reimbursement possible facilitator that could increase *‘behavioural health’* diagnoses^49^ Insurance policies that restrict the number of visits per patient^51^ hamper recognition Difficulties gaining insurance reimbursement for mental health diagnoses^56^ Physical health may sometimes be prioritised as mental health problems are not seen as a *‘chief complaint’*^51^	Lack of time to deal with such [mental health] issues as it is *‘too complicated and difficult’* for the time allowed^54^	Lack of care available from insurance policies^56^ Lack of psychiatrists provided by insurance companies^58^ Limitations on the number of funded therapy visits^57^ Occasional difficulty choosing whether to refer in short appointment times^48^

**Resources**	Lack of tools.^48^^,^^49^^,^^51^^,^^53^^,^^54^ Lack of tools in this area is in contrast to the more extensive availability of tools in the adult mental health field^48^ and for organic illnesses^53^	Desire for more support from other disciplines,^54^ including psychologists, schools, counsellors Collaborating with other groups described as communicating into a *‘void’*,^58^ which results in a separation from available resources	Lack of providers and resources^49^^,^^56^^,^^57^ with practitioners sometimes becoming the *‘“*de facto*” mental health provider*’ as there *‘simply wasn’t anyone else available’* 57 Extensive waiting times for specialists services^50^^,^^54^^,^^56^^,^^58^^,^^59^ Distance to resources was a barrier for rural practitioners^56^ Lack of communication led to a disconnect between primary and secondary care^56^ and *‘contributed to primary care practitioners’ perceptions of poor effectiveness of therapy’* 57 Desire for increased communication,^48^ information,^59^ and feedback on referrals^60^ Dislike of long letters Desire for telephone communication^59^ Frustration with frequent rejection of referrals^50^ Desire for clearer referral criteria — Child and Adolescent Mental Health Services criteria were described as a *‘mystery’* 48 *‘Greater assistance from mental health providers’* was a desired facilitator^49^^,^^56^

**Family issues**	Increased parental awareness of mental health problems was endorsed as a facilitator^49^^,^^51^	*‘A longstanding relationship with the family strengthened the* [practitioner’s] *commitment’* and provided the advantage of contextual knowledge^57^	

**Figure 3.**
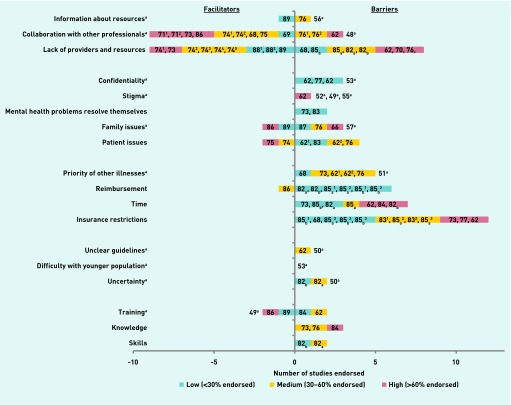
***Undifferentiated barriers. Studies with no colour coding are qualitative (as denoted by the ‘a’) and, as such, level of endorsement does not apply. Superscript numbers mean that the study had more than one item querying this topic and subscript letters are related to the information provided in the Notes column of Table 1.***

Figure 3 provides an overview of the study findings in the undifferentiated category. There was a very apparent desire for collaboration with other professionals and increased providers and resources in the undifferentiated category, with insurance restrictions posing the largest barrier. Qualitative data relating to the undifferentiated barriers are given in Box 1.

Box 1. **Undifferentiated barriers**

**Resources**
- Children’s mental health resources are lacking in comparison with the adult services in terms of collaboration with other professionals,^48^ sometimes resulting in practitioners not being aware of services that may be available to their patients^56^

**Family issues**
- Difficult family circumstances often lead to a lack of appointment uptake^57^
- Confidentiality limitations are a barrier^53^
- Stigma^52^ and negative consequences of labelling^49^^,^^55^

**Prioritisation of mental health problems**
- Reluctance of society to see eating disorders as a serious disease was *‘a severe hindrance’*^51^

**Complexity**
- Uncertainty as to what is expected of practitioners^50^
- Absence of a *‘gold standard’* for dealing with children’s mental health problems, specifically pinpointing *‘unhelpful’* guidelines from the National Institute for Health and Care Excellence^50^
- Relating to young people highlighted as difficult^53^

**Training, knowledge, and skills**
- Lack of training must be addressed as a high priority^49^

## DISCUSSION

### Summary

Primary care practitioners play a crucial ‘gatekeeper’ role to specialist services for children and young people with mental health problems, yet they face numerous barriers, in particular a lack of time, knowledge, reimbursement, mental health providers, and resources. A lack of providers of specialist services was the most highly endorsed barrier overall, with primary care practitioners expressing a clear desire for decreased waiting times and increased resources for referral, particularly in rural areas.^56^^,^^61^^–^64 As all of the facilitators that were identified were the inverse of identified barriers, the discussion focuses on barriers from here on, unless explicitly stated otherwise.

Organising the literature according to stages proved useful as, in some cases, particular barriers applied to some activities but not others; as an example, time restrictions had a *particular* impact on recognition, diagnosis, and management, but not on referral to specialist services. Likewise, insurance restrictions had a particular impact on management and referral to specialist services, but not recognition and diagnosis.

Other barriers that were specific to particular stages included a lack of confidence in identification and diagnosis, along with long waiting times when referring children to specialist services (a reduction in which was the most highly endorsed facilitator overall). Financial concerns were common across all stages but were a particular barrier to managing children with mental health problems within primary care. Notably, although many common issues were seen across different countries, as also found by Vallance *et al*,^91^ all studies that endorsed insurance and reimbursement restrictions were based in the US;^61^^,^^65^^–^71 this highlights the fact that different challenges may arise within different healthcare systems. Barriers in the undifferentiated section provided a more inconsistent picture, possibly due to the fact that the initial questioning was not asked in relation to the specific stages of primary care practitioner management, resulting in primary care practitioners reporting on different things.

### Strengths and limitations

There was wide variability in the quality of included studies, which commonly related to issues with data analysis and poor evidence for the qualitative studies, and issues with the robustness of barrier measures in the quantitative studies. Studies also varied considerably in the extent to which they contributed to the review, with questions about barriers often supplementary to measures focusing on other research questions.

Most studies (*n* = 25) focused on mental health in general, but some highlighted that different sorts of barriers may apply for different types of mental health problems, for example, sleep disorders.^72^

Excluding specific populations, such as those with a primary health diagnosis other than a mental health problem, may limit the generalisability of the review beyond ‘general’ populations. Studies also differed markedly in the age range of children and young people being considered, focusing specifically on pre-adolescen ts,^49^^,^^52^^,^^55^^,^^56^^,^^58^^,^^66^^,^^67^^,^^70^^,^^73^^–^76 adolescents,^50^^,^^53^^,^^57^^,^^61^^,^^77^^,^^78^ or a combination of the two,^48^^,^^51^^,^^54^^,^^59^^,^^60^^,^^62^^–^65^,^^68^^,^^69^^,^^71^^,^^72^^,^^79^^–^91 limiting the extent to which the needs of each group can be identified.

The exclusion of studies published in a language other than English limits the scope of this review and must be taken into account when considering to which countries these results are applicable.

Finally, given that identification of mental health problems in children and young people has been found to be low in primary care practitioner settings,^32^ it is important to note that all the studies included in this review used self-report measures of barriers and, as such, cannot provide any information about barriers in situations where primary care practitioners have failed to identify a mental health problem.

The review does have some limitations. The search strategy used online databases, which would not capture unpublished material. Barriers and facilitators were also defined in a way that did not include primary care practitioners’ perceptions of responsibility, confidence, and satisfaction unless they had specifically endorsed these as being an obstacle or desired change. Furthermore, studies did not always explicitly label ‘barriers’ and ‘facilitators’, and, as such, interpretation was needed in some cases.

Particular strengths of the review include the incorporation of both qualitative and quantitative research and the division of the barriers into diagnosis and treatment phases to allow a clearer look at specific issues in primary care. In addition, a rigorous, systematic method was used, which involved the use of two raters at every stage, abstract and full-text screen, data extraction, and quality assessment. A third rater was brought in whenever disagreements occurred, strengthening the objectivity of the process.

### Implications for research and practice

Further research is required to identify the specific challenges faced by primary care practitioners at different stages from identification to referral to specialist services, for specific mental health problems, and with particular patient populations (for example, young–older children, rural– urban settings). Given the lack of research in this area, mixed-methods approaches will be valuable to explore patients’ and primary care practitioners’ perspectives, quantify the extent to which particular barriers influence management, and identify the circumstances in which these barriers apply. These findings can then be used to target strategies to improve access to good-quality mental health care among children and young people. Future research should also aim to develop measurements that are more robust, as it is clear that there is a need for more rigour in the design and analysis of barrier measures.

Primary care practitioners identified and endorsed a wide range of barriers that prevent them from effectively supporting children and young people with mental health problems, reflecting a need for improvements.^92^ The most obvious improvement is the need for more resources and providers of mental health services for children and young people in order to reduce waiting times and improve access to specialist services. Better access would also be facilitated, at least in part, by increased communication and collaboration with these services.

Primary care practitioners also clearly identified a lack of confidence in recognising childhood mental health problems and a lack of training in this area, which, given the prevalence of such issues,^3^ is resulting in a serious skill gap. The development of appropriate and evidence-based screening tools for common mental health problems for use in primary care, as already exists for adults,^93^ would be a positive step to rectify this situation.

Given the time restrictions that primary care practitioners experience, they often do not consider themselves to be in a position to manage childhood mental health problems but desire increased collaboration with other professionals. The introduction or expansion of primary-care-based mental health services would relieve the pressure on primary care practitioners and allow quicker access to evidence-based interventions. The integration of primary and secondary services is challenging within some healthcare systems due to funding arrangements (for example, in the UK)^94^ and changes at policy levels may be required to promote increased collaboration.^22^ However, there are good examples of effective collaborative care models for managing adult mental health problems.^95^ A recent systematic review has provided evidence supporting the effectiveness of integrated medical behavioural primary care for improving youth mental health outcomes^96^ in which various integration models were reviewed. The results emphasised that those trials that used a collaborative care model produced the largest effect sizes.

Given the high prevalence and significance of mental health problems in children and young people, it is clear that serious attention is required to support primary care practitioners in facilitating access to evidence-based interventions and greater resources.

## Appendix 1. Search terms

(i) (primary care OR general practi* OR pediatrician OR paediatrician)

AND

(ii) (anxi* OR suici* OR affec* OR psychosis OR self-harm OR mental OR depress* OR disorder* OR externali* OR internali* OR oppositional OR conduct OR ADHD)

AND

(iii) (child* OR youth* OR adolescen*)

AND

(iv) (barrier* OR access* OR service* OR recogni* OR “unmet need” OR refer* OR manag*) NOT dent* NOT oral* NOT infect* NOT immun*)

Limited to “article”, “English”, and searched “title & abstract”

## Appendix 2. Data extraction template

**Table:**

Author	Year	Title	Journal	Study characteristics			Sample/participant characteristics								Study aim	Measure of perceived barriers/facilitators	Barrier reported	% endorsed	Facilitator reported	% endorsed
							Number of partcipants (PCPs)	Age (mean)	Sex (% female)	Age focus	Child specialist	Practitioner type	Setting	Mental health focus						
